# Novel risk factors for primary prevention of oesophageal carcinoma: a case-control study from Sri Lanka

**DOI:** 10.1186/s12885-018-4975-4

**Published:** 2018-11-19

**Authors:** Ishanka Ayeshwari Talagala, Metthananda Nawarathne, Carukshi Arambepola

**Affiliations:** 1 National Programme for prevention and control of non-communicable diseases; Ministry of Health, Nutrition and Indigenous Medicine, Colombo, Sri Lanka; 2 National Hospital, Colombo, Sri Lanka; 3 Department of Community Medicine, Faculty of Medicine, University of Colombo, Colombo, Sri Lanka

**Keywords:** Oesophageal carcinoma, Novel risk factors, Primary prevention, Sri Lanka

## Abstract

**Background:**

Oesophageal carcinoma (OC) is one of the leading cancers in Sri Lanka. Its increasing incidence despite the implementation of various preventive activities addressing the conventional risk factors indicates the possibility of the existence of novel, country-specific risk factors. Thus, the identification of novel risk factors of OC specific to Sri Lanka is crucial for implementation of primary prevention activities.

**Methods:**

A case-control study was conducted among 49 incident cases of OC recruited from the National Cancer Institute, Maharagama using a non-probability sampling method, and unmatched hospital controls (*n* = 196) excluded of having OC recruited from the endoscopy unit of the National Hospital of Sri Lanka. Data were collected using an interviewer administered questionnaire. Risk factors for OC were assessed by odds ratio (OR) with 95% confidence interval (CI). The risk factors were adjusted for possible confounding by logistic regression analysis.

**Results:**

Of the study population, OC was common among males (69%) and the majority presented with squamous cell carcinoma (65%) at late stages (Stage IV: 45%; Stage III: 37%). Following adjusting for confounders, the risk factor profile for OC included; age > 65 years (OR = 4.0; 95% CI: 1.2–14.2); family history of cancer (OR = 5.04; 95% CI: 1.3–19.0); sub-optimal consumption of dietary fibre (OR = 3.58; 95% CI: 1.1–12.3); sub-optimal consumption of anti-oxidants (OR = 7.0; 95% CI: 2.2–22.5); over-consumption of deep fried food (OR = 6.68; 95% CI:2.0–22.6); ‘high risk’ alcohol drinking (OR = 11.7; 95% CI: 2.8–49.4); betel quid chewing (OR = 6.1; 95% CI: 2.0, 20.0); ‘low’ lifetime total sports and exercise activities (MET hours/week/year) (OR = 5.83; 95% CI: 1.5–23.0); agrochemicals exposure (OR = 6.57; 95% CI: 1.4–30.3); pipe-borne drinking water (OR = 5.62; 95% CI:1.7–18.9) and radiation exposure (OR = 4.64; 95% CI: 1.4–15.5). Significant effect modifications were seen between betel quid chewing and male sex (*p* = 0.01) and between ever exposure to radiation and age over 65 years (*p* = 0.04).

**Conclusions:**

Risk profile for OC includes novel yet modifiable risk factors in relation to diet, occupation, environment and health. Primary prevention should target these to combat OC in Sri Lanka.

**Electronic supplementary material:**

The online version of this article (10.1186/s12885-018-4975-4) contains supplementary material, which is available to authorized users.

## Background

Cancer plays a major role in the global burden of diseases. In particular, oesophageal carcinoma signifies a disease of public health importance as the eighth commonest cancer in the world [1]. Despite the recent advances in clinical management [2], it shows poor prognosis with only 5–10% five-year survival, contributing to all cancer deaths by 6% and assuming the sixth leading cause of cancer deaths worldwide [1]. It is also the sixth leading cause for cancer related years of life lost (YLL) in the world [3] with YLL accounting for 97% of the total disability adjusted life years (DALYs) [4]. More importantly, oesophageal cancer has been identified as a virulent tumour in developing countries; assuming the fourth place among all cancers, comprising 81% of all newly diagnosed oesophageal carcinoma cases in the world, and 84% of DALYs attributable to cancers occurring in the developing countries [4]. According to the latest statistics, the highest incidence has been reported from Asia and Africa [1], thus necessitating prompt action for prevention in these regions.

Sri Lanka, a developing country located in South Asia reports oesophageal cancer as the third commonest cancer following breast cancer and cancers of the oral cavity [5]. It has continued to be the third commonest cancer among males and fifth commonest among females [5]. With the middle-age population being more vulnerable to this disease, it has affected the national labour force [5, 6]. Also, with cancer care being predominantly provided by the state, and increasingly financed by out of pocket spending [7], it poses a great impact on the economy of individuals, households as well as the country.

Considering the importance of combating cancers in the world including oesophageal carcinoma, ‘World Cancer Declaration’ endorsed by the World Cancer Congress in 2010 calls upon all the member states of the World Health Organization to implement nine immediate actions including policy development, cancer prevention and early detection by strengthening health systems [8]. The National policy and strategic framework on cancer prevention and control, Sri Lanka - 2015, thus identifies “Ensuring primary prevention of cancers by addressing risk factors and determinants by improved public awareness and empowerment” as a policy objective and implement various activities in order to prevent any type of cancer by combating its risk factors [9]. However, despite oesophageal carcinoma being one of the leading cancers in Sri Lanka, there is no specific prevention programme implemented against it in the country.

Prevention of oesophageal carcinoma is based on the principles of primary prevention on reducing the burden of its risk factors. The underlying causes of oesophageal carcinoma are multi-factorial. As with other chronic non-communicable diseases, the commonly known risk factors are related to one’s non-modifiable demographic (e.g. older age, male sex, genetic predisposition [1, 10]), socio-economic and clinical status (e.g. low socio-economic status [11–13], gastro-oesophageal reflux disease [14], Barrett’s oesophagus [15], non-steroidal anti-depressants (NSAIDs)/Aspirin [16]) and also to modifiable lifestyle related factors (e.g. physical inactivity [17], tobacco, alcohol, low intake of fruits and vegetables [11, 18]).

It is shown that the incidence of oesophageal carcinoma in Sri Lanka has been gradually yet steadily increasing in the recent past. This increase may be due to an increasing trend of adenocarcinoma which shows a more aggressive disease progression [5, 6, 19–21] than the squamous cell carcinoma which is the commonest histology type (73% of cases) [5]. Another important reason could be the increased susceptibility owing to increasing trends in the prevalence of risk factors related to lifestyle. It is shown that along with rapid and unplanned urbanization in the last few decades, several unhealthy lifestyle practices have become prominent, such as fruit and vegetable intake being far below the recommended level in 73% [22]; prevalence of alcohol consumption in urban settings being 30% [23]; prevalence of current alcohol drinking being 18%, percentage of current smoking being 15%; and overall prevalence of inactivity being 30% [22] in Sri Lanka. More importantly, the increasing trends in oesophageal cancer could also be attributable to not yet identified risk factors that are socio-culturally and environmentally unique to countries within a region.

In the recent past, several risk factors have been newly identified related to specific elements in diet (e.g. diet deficient in vitamins A, B, C and E, Selenium and Beta carotene [24, 25]; food preparation methods (e.g. chillies [24, 26] and spicy food [27]); food consumption patterns (e.g. consuming food/drinks at high temperatures [27]); drinking water sources [28]; daily habits (e.g. chewing betel leaf with tobacco [29] and arecanut [30]); and exposure to ionizing radiation [31]. However, almost all these risk factors have been identified in studies carried out among populations in developed countries, and therefore may not directly apply to the risk profile relevant to populations in developing countries. Furthermore, unlike developed countries, most of the developing countries are agriculture based, and thereby exposed to indiscriminate use of agrochemicals and other occupational hazards. Currently, there is minimal research evidence on its risk for oesophageal carcinoma.

Therefore, in order to prioritize primary prevention strategies in low resource settings as in Sri Lanka, it necessitates a detailed assessment of population-specific risk factor profiling. Identification of such specific risk profile for the country would enable the health care planners to prioritize the risk factors that ought to be addressed in primary prevention, and it would be applicable to other developing countries especially in low-resource settings. This study was conducted with the aim of determining the magnitude of risk factors of oesophageal carcinoma among adults in Sri Lanka.

Additional file 1 gives the details of this manuscript being reported based on the STROBE guidelines for reporting observational studies [32].

## Methods

A hospital based unmatched case-control study was conducted among residents in the Western Province during 2015–2016 in Sri Lanka.

### Selection of cases

Cases were newly diagnosed patients of oesophageal carcinoma based on histological confirmation made during last 3 months following upper gastro-intestinal endoscopy (UGIE) examination. The authors recruited the cases consecutively from surgical and oncology wards and clinics of the National Cancer Institute, Maharagama (NCIM), which is the premier tertiary referral hospital in Sri Lanka dedicated for treatment and follow up of cancer patients referred from both state (predominantly) and private sector hospitals. It is the only hospital providing in-ward and out-patient care services including chemotherapy and radiotherapy for residents in the Western Province. Critically ill patients, patients with secondary carcinoma (e.g. metastasis) including oesophageal carcinoma, patients diagnosed with any other cancer (confirmed with documental evidence) and patients with relapses of any cancer including oesophageal carcinoma were excluded from the study.

### Selection of controls

Controls were persons excluded of oesophageal carcinoma based on UGIE examination findings. The authors of the study recruited the control group based on the incidence density sampling method (i.e. four controls selected within 1 week of a case recruitment) [33], from the endoscopy unit of the National hospital of Sri Lanka, which is the leading referral unit for patients from state and private sector hospitals for high risk screening (e.g. family screening) and diagnosis of cancer. Patients diagnosed of having any cancer, patients referred for high risk screening for oesophageal carcinoma (e.g. family history), patients with cirrhosis/chronic liver diseases, patients having any oesophageal/gastric structural abnormalities on UGIE such as polyps, ulcers and strictures, patients diagnosed with conditions that may mask visualization of the oesophageal mucosa (e.g. oesophagitis, gastro-oesophageal reflux disease, Barrette’s oesophagus, achalasia and/or hiatus hernia) and patients with a history of dyspeptic symptoms persisting for more than six months were excluded from the study. All these exclusions were confirmed by documental evidence and by performing an UGIE examination by two Consultant Gastroenterologists, based on the visualization of apparently healthy oesophageal mucosa, gastro-oesophageal junction and the stomach up to the distal duodenal sphincter with no macroscopic changes, erosions or lesions such as polyps and ulcers.

Controls for the study could not be recruited from the endoscopy unit at NCIM as it provides services mainly for treatment purposes, screening for secondary oesophageal carcinoma and for high risk screening (e.g. family screening), and very rarely for diagnostic purposes of primary oesophageal carcinoma. This selection bias is however assumed to be low as both NCIM and National Hospital of Sri Lanka were comparable by both being tertiary care level state hospitals with similar patient characteristics.

### Sample size calculation

The sample size for case (*n* = 49) and control groups (*n* = 196) for univariate analysis was calculated considering four controls per case, 5% significance level, beta error of 0.2 and 5% non-response rate to detect the smallest risk (odds ratio of 2.5 for consumption of alcohol on oesophageal carcinoma [34] and 33% prevalence of the risk factor among the community controls [35] in Sri Lanka.

### Study variables

Following informed written consent, case and control groups were administered a questionnaire to collect data on socio-demographic characteristics and potential risk factors of oesophageal carcinoma from both case and control groups. These included factors related to personal [e.g. age, sex, ethnicity, level of education, social class (classification based on the occupation [36]) and family history]; occupational and environmental factors (e.g. exposure to agrochemicals, home/industry based chemicals, in-door and out-door air pollution, major source of drinking water); health related factors (e.g. prolonged medication and co-morbidities, exposure to ionizing radiation) and lifestyle related factors (e.g. diet quality and physical activity, alcohol and tobacco use, food/beverages consumed at high temperatures, consumption of coloured beverages and/or spicy food and betel quid chewing).

### Validated questionnaires used in the study

Two already developed and validated questionnaires were used in the current study to assess the quality of the diet and the physical activity of the study participants in both groups. The quality of the diet related to cancer was assessed using a food frequency questionnaire (FFQ), which was developed and validated for assessing the quality of diet in relation to cancer in the district of Colombo [37]. Lifetime total physical activity was assessed using lifetime total physical activity questionnaire which has been validated [37], and used worldwide including in Sri Lanka.

Data related to other study variables were obtained through a pre-tested interviewer administered questionnaire developed by the authors. The judgemental validity of the developed questionnaire was assessed by an expert panel including Oncologists, Onco-surgeons, Consultant Community Physicians, and Psychiatrists. Operational definitions used for each variable in the study are given in Table 1.

**Table 1:** Operational definitions used in the study

Term	Definition used in the study
‘High risk’ drinker	A person who has consumed more than three standard drinks on any day or more than seven standard drinks per week for women, and more than four drinks on any day or more than 14 per week for men [62]. Standard drink: Quantity of any type of drink, containing 10 grams of pure alcohol [62, 63]. *The volume of alcoholic beverage consumed per session was based on standard volume measures for different types of alcoholic beverages consumed (e.g. glass, shots, bottles) and the total volume of ethanol consumed per session per week was calculated using the standard ethanol amount in the drinks* [64].
‘Sub-optimal’ consumption of fibre and antioxidants	Inadequate consumption of food containing dietary fibre and anti-oxidants [37].
‘Sub optimal’ consumption of deep fried food	Overconsumption of deep fried food [37].
Consumption of beverages at extreme temperatures	Daily consumption of hot beverages (tea/coffee) within at least 2 minutes of preparation
Consumption of excessive chilli	Daily consumption of meals prepared with excessive additional chilli
Food/beverages with excessive colour	Any coloured food/beverage that would stain the fingers, lips and tongue upon its consumption
‘Low’ and ‘high’ total lifetime physical activity levels	Lifetime total physical activity (average MET-hours per week per year) in relation to occupation, household and sports and exercise activities [37, 65]. ‘Low’ level of physical activity = the score that corresponded with the 25^th^ percentile and ‘high’ level of the physical activity = the score that corresponded with the 75^th^ percentile of the distribution of total scores of all participants
Ever smoker	A person who had smoked 100 or more cigarettes/sticks of bidi in his/her lifetime [66].
Exposure to passive smoking	A person who has/had been exposed to second hand smoking in confined areas (at home or work) for at least once a week for three consecutive months during the past one year
Ever betel chewer	A person who has/had the habit of betel quid chewing (with or without additives) at least three times per week, continuously for six months or more in the lifetime
Exposure to agrochemicals	Direct and/or indirect exposure to agrochemicals Where; ‘Direct exposure’ to agrochemicals = Persons who have been farmers/cultivators for at least six months during which they have been involved in spraying/handling agrochemicals And ‘Indirect exposure’ to agrochemicals = Persons who have been living at least for six months in close proximity (within 100m) and/or working in close proximity (10m) to an environment consistently exposed to agrochemicals
Exposure to home/industry based chemicals	Direct and/or indirect exposure to home/industry based chemicals Where; Direct exposure = persons who have been working at least six months during which they were directly exposed to home/industry based chemicals for at least three days per week And Indirect exposure = Workers who have not been directly handling chemicals, but have been working for at least six months in close proximity (10m radius) to an environment consistently using home/industry based chemicals
Major source of drinking water	The source of drinking water from which he/she consumed water consistently at least once a day, during the last one year. If there were more than one source (eg: pipe borne water consumption at the work place and consumption of well water at home), most frequently used source of drinking water was considered
Exposure to indoor air pollution	Use of fire wood as the main source of fuel in the household and having the kitchen built attached to the house
Exposure to outdoor air pollution	Person who has been living at least for six months in a place adjacent (within 20m) to a main road and/or living at least for six months in close proximity (within 100m radius) to a factory with smoke emission
Prolonged medication for co-morbid conditions	A person who had been on a drug regime (oral medications) for at least six consecutive months, during the last 10 years
Vitamin supplementation	A person who had been on vitamin supplementation for at least one month consecutively during the last 10 years
Exposure to ionizing radiation	A person who has been exposed to any type of ionizing radiation (eg: X-ray, CT, Gamma rays) within the past 10 years irrespective of the indication

### Data analysis

Data were analysed using the statistical package for the social sciences (SPSS) version 20. Descriptive statistics with mean (SD) for continuous data and proportions for categorical data were used respectively. The risk of each factor in the development of oesophageal carcinoma was assessed using odds ratio (OR) with 95% confidence interval. These ORs were adjusted for confounders by carrying out logistic regression (LR) analysis using backward LR method, based on two assumptions: the sample size was adequate for logistic regression analysis (at least 10 cases per independent variable tested) and there was no multicollinearity between the predictor variables [38]. The dependent variable in the LR model was presence or absence of oesophageal carcinoma. Variables that showed a significant association with oesophageal carcinoma at a significance level of 0.05 were included as independent variables into the model. As pre-decided by an expert panel, having less than 10 in the control or case groups [38], variables that were strongly correlated with other variables, variables showing a large confidence interval in the univariate analysis with an upper limit of more than 30, and variables that were not meaningful and plausibly explained by the model as risk factors were excluded from the LR. Goodness of fit of the LR model was assessed by the overall percentage of prediction of oesophageal carcinoma by the model, chi-square test, Hosmer and Lameshow test, Omnibus test, Cox and Snell Square test and Negelkerke R^2^ tests.

In addition, variables showing significant associations with oesophageal carcinoma were further tested for possible effect modification of the risk for oesophageal carcinoma, and if present, such interactions were also included in the LR analysis.

## Results

The sample consisted of 49 cases and 196 controls. The response rate was 100%. The mean age of cases was 59.8 (SD = 11.2) years ranging between 37 and 79 years, while the mean age of controls was 53.0 (SD = 15) ranging between 18 and 87 years. The standardized skewness for age in the study was 2.47 and the standardized kurtosis was 1.31. Other basic characteristics of the cases and controls are given in Table 2.

**Table 2:** Distribution of the demographic characteristics of cases and controls

Demographic characteristic	Cases (*N* = 49)		Controls (*N* = 196)	
	No.	%	No.	%
Sex				
Male	34	69.4	70	35.7
Female	15	30.6	126	64.3
Current marital status				
Married	41	83.6	136	69.4
Single	4	8.2	39	19.8
Divorced/separated/widowed	4	8.2	21	10.8
Ethnicity				
Sinhala	43	87.8	145	74.0
Other ethnicities (Tamil, Muslim, Burgher)	6	12.2	51	26.0
Highest educational level				
Primary and lower	15	30.6	31	15.8
Secondary	33	67.4	153	78.0
Tertiary	1	2.0	12	6.2
Employment status				
Currently employed	7	14.2	72	36.7
Previously employed	35	71.4	59	30.1
Never employed	7	14.3	65	33.2
Social status^a^				
Social class I-Leading professionals	1	2.0	8	4.1
Social class II-Lesser professionals	2	4.1	18	9.2
Social class III-Skilled workers & non-manual workers	15	30.6	56	28.6
Social class IV- Partly skilled workers	13	26.5	28	14.3
Social class V- Unskilled workers^b^	18	36.7	86	43.9
Monthly family income (SLR)				
< 20,000	24	49	88	44.9
20,001-50,000	20	40.8	90	45.9
50,001-100,000	5	10.2	15	7.7
> 100,000	0	0.0	3	1.5

^a^Classification based on the occupation

^b^Based on the classification, the unemployed are included in the social class V as well

At the time of diagnosis, 85.7% of cases and 63.3% of controls were unemployed, and 36.7% of cases and 43.9% of the controls belonged to social class V (unskilled labourers, unemployed individuals). Majority of the cases had squamous cell carcinoma (65.3%); located in the lower thoracic oesophagus (61.2%); in stage IV (45%) and stage III (37%) of the disease; with moderately differentiated cells (65.3%). All cases (100%) had sought medical attention following progressive dysphagia, with a mean duration of 2.9 months (SD = 1.4) since the development of first symptom to the diagnosis, with a range of 1–6 months. Also, at the time of diagnosis, most cases (55.1%) had been suffering from regurgitation for a mean duration of 1.2 years (SD = 1.4); 49% from nausea for a mean duration of 0.78 years (SD = 1.1); 47% from heart burn for a mean duration of 0.85 years (SD = 1.2); 43% from loss of appetite for a mean duration of 0.7 years (SD = 1.1); and 37% from vomiting for a mean duration of 0.54 years (SD = 0.8), which they had not attributed to oesophageal carcinoma.

The majority of controls however, were having dyspeptic symptoms (98%) including complaints of dysphagia (2%), for which they sought medical attention. On the other hand, controls with a history suggestive of long standing (more than 6 months) dyspeptic symptoms were excluded in the current study and oesophageal carcinoma was excluded following UGIE among them.

Of the several variables that showed significant OR for oesophageal carcinoma in the univariate analysis (Tables 3, 4 and 5), 19 were selected for the LR analysis. Based on these results, the country-specific risk profile for oesophageal carcinoma included: age more than 65 years; family history of cancer; sub-optimal consumption of fibre; sub-optimal consumption of antioxidants; over-consumption of deep fried food; ‘low’ total lifetime sports and exercise activities; ‘high risk’ alcohol consumption; ever betel quid chewing; exposure to agrochemicals; consuming pipe-borne water as the major source of water and ever exposure to radiation. The final LR model was able to classify the cases from controls with 90.6% accuracy compared to 80% without any of the independent variables used in the model. According to Cox and Snell R square and Nagelkerke R square test results, the model described 45.3–71.7% of the variability of the dependent variable by the independent variables. Results of Hosmer and Lemeshow test [38] were; X^2^ = 4.63; df = 8; *p* = 0.796. Sensitivity of the model was 67.3%, while the specificity was 96.4%. The positive and negative predictive values of the model were 82.5% and 92.2% respectively.

**Table 3:** Risk of oesophageal carcinoma associated with demographic, family history and socio-economic characteristics

Characteristic	Cases (*N* = 49)		Controls (*N* = 196)		Non-adjusted OR (95% CI)	Adjusted OR (95% CI)
	No.	%	No.	%		
Age						
> 65 years	17	34.7	41	20.9	**2.0 (1.2–4.0)**	**4.0 (1.2–14.2)**
< 65 years (reference)^a^	32	65.3	155	79.1	1.0	1.0
Sex						
Male	34	69.4	70	35.7	**4.1 (2.1–8.0)**	NS^b^
Female (reference)^a^	15	30.6	126	64.3	1.0	
Family history of cancer						
Yes	11	22.4	20	10.2	**2.6 (1.1–5.8)**	**5.0 (1.3–19.0)**
No (reference)^a^	38	77.6	176	89.8	1.0	1.0
Employment status						
Ever employed^d^	42	85.7	132	67.4	**2.9 (1.2–6.8)**	NS^b^
Never employed (reference)^a^	7	14.3	64	32.6	1.0	
Social status						
Low (social class III, IV & V)	46	93.9	170	86.7	2.35 (0.7–8.1)	–
High (social class I & II) (reference)^a^	3	6.1	26	13.3	1.0	
Monthly family income (SLR)						
< 20,000	24	49.0	88	44.9	1.2 (0.6–2.2)	–
> 20,000 (reference)^a^	25	51.0	108	55.1	1.0	

^a^The category having the lesser proportion of cases

^b^Not significant in the logistic regression model

^c^Previously employed & currently employed categories were amalgamated for univariate analysis

**Table 4:** Risk of oesophageal carcinoma associated with the modifiable life style related factors

Characteristic	Cases (*N* = 49)		Controls (*N* = 196)		Non-adjusted OR (95% CI)	Adjusted OR (95% CI)
	No.	%	No.	%		
Dietary fibre						
Sub-optimal	22	44.9	15	7.7	**9.83 (4.6–21.2)**	**3.58 (1.1–12.3)**
Optimal (reference)^a^	27	55.1	181	92.3	1.0	1.0
Anti-oxidants						
Sub-optimal	32	65.3	44	22.4	**6.5 (3.3–12.8)**	**7.0 (2.2–22.5)**
Optimal (reference)^a^	17	34.7	152	77.6	1.0	1.0
Deep fried food						
Sub-optimal^b^	26	53.1	44	22.4	**3.91 (2.0–7.5)**	**6.68 (2.0–22.6)**
Optimal (reference)^a^	23	46.9	152	77.6	1.0	1.0
Beverages (tea/coffee) at extremely hot temperatures						
Yes	21	42.9	9	4.6	**15.6 (6.5–37.4)**	Excluded from LR^c^
No (reference)^a^	28	57.1	187	95.4	1.0	
Meals containing excessive amount of chillies						
Yes	36	73.5	15	7.7	**33.4 (14.7–76.2)**	Excluded from LR^c^
No (reference)^a^	13	26.5	181	92.3	1.0	
Food/drink with excessive colouring						
Yes	40	81.6	34	17.3	**21.2 (9.4–47.7)**	Excluded from LR^c^
No (reference)^a^	9	18.4	162	82.7	1.0	
Alcohol consumption						
Yes	30	61.2	45	23.0	**5.3 (2.7–10.3)**	Excluded from LR^c^
No (reference)^a^	19	38.8	151	77.0	1.0	
Risk drinking category						
High risk drinking	27	55.1	18	9.2	**12.14 (5.8–25.5)**	**11.7 (2.8–49.4)**
Low risk drinking^d^	3	6.1	27	13.8		
never consumers^d^ (reference)^a^	19	38.8	151	77.0	1.0	1.0
Smoking status						
Ever smoker	28	57.1	21	10.7	**11.1 (5.4–22.9)**	NS^e^
Never smoker (reference)^a^	21	42.9	175	89.3	1.0	
Exposure to passive smoking						
Yes	32	65.3	24	12.2	**13.49 (6.5–27.9)**	NS^e^
No (reference)^a^	17	34.7	172	87.8	1.0	
Betel quid chewing						
Ever chewed	31	63.3	21	10.7	**14.35 (6.8–30.0)**	**6.1 (2.0–20.0)**
Never chewed (reference)^a^	18	36.7	175	89.3	1.0	1.0
Lifetime total occupational activities (MET hours/week/year)^f^						
Low (< 1.0)	10	20.4	66	33.7	0.51 (0.2–1.1)	NS^e^
Average (1.0–218.1)^g^	17	34.7	91	46.4		
High (> 218.1)^a^ (reference)^g^	22	44.9	39	19.9	1.0	
Lifetime total household activities (MET hours/week/year)^f^						
Low (< 40.7)	26	53.1	35	17.9	**5.2 (2.7–10.2)**	NS^e^
Average (40.7–323.8)^h^	21	42.9	104	53.1		
High (> 323.8)^h^ (reference)^a^	2	4.1	57	29.1	1.0	
Lifetime total sports and exercise activities (MET hours/week/year)^f^						
Low (< 1.0)	39	79.6	111	56.6	**2.99 (1.4–6.3)**	**5.83 (1.5–23.0)**
Average (1.0–40.0)^i^	5	10.2	30	15.3		
High (> 40.0)^i^ (reference)^a^	5	10.2	55	28.1	1.0	1.0

^a^The category having the lesser proportion of cases

^b^Sub-optimal consumption of deep fried food indicates over consumption levels

^c^Excluded from the LR analysis due to large confidence interval (upper limit being > 30.0)

^d^Low risk drinking and never consumer categories were amalgamated for univariate analysis

^e^Not significant in the logistic regression model

^f^The highest and the lowest quartiles of the activity were considered as the cut off points

^g,h,i^Average and high levels of physical activity categories were amalgamated for univariate analysis

**Table 5:** Risk of oesophageal carcinoma associated with occupational, environmental and health related factors

Characteristic	Cases		Controls		Non-adjusted OR (95% CI)	Adjusted OR (95% CI)
	No.	%	No.	%		
Exposure to agrochemicals						
Ever exposed	19	38.8	19	9.7	**5.9 (2.8–12.4)**	**6.57 (1.4–30.3)**
Never exposed (reference)^a^	30	61.2	177	90.3	1.0	1.0
Exposure to other home/industry based chemicals						
Ever exposed	11	22.4	9	4.6	**6.0 (2.3–15.5)**	NS^b^
Never exposed (reference)^a^	38	77.6	187	95.4	1.0	
Major source of drinking water						
Pipe borne water	27	55.1	65	33.2	**2.47 (1.3–4.7)**	**5.62 (1.7–18.9)**
Ground water (reference)^a^	22	44.9	131	66.8	1.0	1.0
Indoor air pollution						
Yes	39	79.6	18	9.2	**38.6 (16.5–90.0)**	NS^b^
No (reference)^a^	10	20.4	178	90.8	1.0	
Outdoor air pollution						
a. **Factory in close vicinity**						
Yes	4	8.2	16	8.2	1.0 (0.32–3.1)	–
No (reference)^a^	45	91.8	180	91.8	1.0	
b. **House situated adjacent to a main road**						
Yes	20	40.8	53	27.0	1.86 (1.0–3.6)	–
No	29	59.2	143	73.0	1.0	
On prolonged medication						
Yes	4	8.2	13	6.6	1.25 (0.4–4.0)	–
No (reference)^a^	45	91.8	183	93.4	1.0	
On vitamin supplementation						
Yes	1	2.0	3	1.5	1.34 (0.1–13.2)	–
No (reference)^a^	48	98.0	193	98.5	1.0	
Exposure to radiation						
Ever exposed	19	38.8	19	9.7	**5.9 (2.8–12.4)**	**4.64 (1.4–15.5)**
Never exposed (reference)^a^	30	61.2	177	90.3	1.0	1.0

^a^The category having the lesser proportion of cases

^b^Not significant in the logistic regression model

Two significant effect modifications between betel quid chewing and male sex (*p* = 0.01), and between ever exposure to radiation and age over 65 years (*p* = 0.04) were also observed. Accordingly, a male with ever betel quid chewing showed a 24-fold risk (OR = 23.9) for oesophageal carcinoma than a male who did not. For a female, this risk was four-fold (OR = 3.9). Also, an individual of over 65 years with ever exposure to radiation had a 24-fold risk (OR = 23.6) for oesophageal carcinoma compared to an individual with no such exposure. For an individual less than 65 years old, this risk was only four-fold (OR = 3.8). However, when the model was re-tested with these effect modifications, those lost their significance.

## Discussion

This study aimed at assessing the population-specific risk factor profile for oesophageal cancer in Sri Lanka in order to supplement the implementation of more targeted preventive programmes for those at risk in order to combat the disease. Our study highlights that the country specific risk factor profile for oesophageal cancer consists of novel risk factors related to lifestyle (sub-optimal consumption of fibre, antioxidants and deep fried food, low total lifetime sports and exercise activities, ever betel quid chewing); environment and occupation (exposure to agrochemicals, pipe-borne drinking water); and health (ever exposure to radiation), in addition to the conventional (age > 65 years, family history of cancer, high risk alcohol consumption) risk factors. The high risk for oesophageal carcinoma usually associated with conventional risk factors such as male sex and tobacco smoking were not apparent in this study.

Considering the country specific risk factor profile that was identified in the current study, it is important to note that many of these risk factors are shared with other cancers as well. For example, increased age, family history of cancer, low fruits and vegetable consumption resulting in low consumption of fibre and anti-oxidants, over consumption of deep fried food, alcohol consumption, betel quid chewing, physical inactivity and tobacco smoking are shared as risk factors with oral cancer, lung cancer, cervical cancer and breast cancer; some of the most common cancers among Sri Lankan males and females [39]. The National Cancer Control Programme of Sri Lanka should therefore build on the initiatives already taken to improve the risk related to other cancers, such as public awareness programs and empowerment through community based health promotion. In addition, there are regulations related to prohibition of smoking in public places; tobacco advertising, promotion and sponsorship; having 80% pictorial warnings on tobacco package [40] implemented in Sri Lanka. Also, manufacturing, importing, selling or offer to sale of any smokeless tobacco products or mixture of any flavoured, coloured or sweetened cigarettes or electronic cigarettes that contain tobacco is prohibited in Sri Lanka. Increased taxation of tobacco and alcohol, prohibition of selling tobacco to children less than 21 years and alcohol to less than 18 years are a few other legislations undertaken to limit the affordability, availability and delay the age of initiation [40]. These should be strictly enforced in the country.

Optimal consumption of dietary fibre and antioxidants is known to lower the risk of oesophageal carcinoma [41, 42]. This relationship is reinforced in the current study, while highlighting several implications in countries having tropical climates such as in South East Asia, West Africa, Central Africa and the Caribbean, which are rich in tropical fruits, vegetables, pulses and legumes. Most of these foods are home grown in the dry zone in Sri Lanka. Therefore, options should be explored collectively by the agricultural sector and the health planners in encouraging a diet rich in fruits and vegetables as a preventive strategy for oesophageal carcinoma.

In developing countries, urbanization, industrialization and economic transition has resulted in a shift of the usual dietary patterns of the people from fruits and vegetable based diet to increased consumption of processed food; from home-cooked food to take-away fast foods; and increased use of fats and sugar sweetened beverages [43]. In Sri Lanka, unhealthy dietary behaviour related to high consumption of deep fried food and added sugar is well established in urban areas [44] and fast spreading to the rural areas. In this background, the consumption of energy dense, deep fried food will further increase the risk of oesophageal cancer [45] and therefore, specific preventive strategies such as effective implementation of the healthy canteen policy in schools [46] and at work places; nutrient profiling; traffic light front of pack food labelling and limiting advertising of such energy dense and sugar sweetened food/beverages to children during the prime television viewing times should be undertaken in the country to combat these risk factors.

There are many studies that show a positive relationship between obesity and oesophageal carcinoma, predominantly for adenocarcinoma [47, 48]. Lagergren et al. (1999) showed that the risk of obese persons [Body Mass Index (BMI) > 30 kg/m^2^] for oesophageal carcinoma compared to those with BMI < 22 kg/m^2^ was 16-fold (OR = 16.2; 95% CI: 6.3, 41.4) [47]. However, in the present study, overweight and obesity (BMI > 25 kg/m^2^) showed potentially an inverse relationship with the risk of oesophageal carcinoma (unadjusted OR = 0.07, 95% CI: 0.02–0.2). Oesophageal carcinoma is a common disease to lose weight due to its disease process. The anthropometry measurements in the current study were taken at the time of recruitment, after the onset of the disease, by which time a higher proportion of cases had lost their weight. Therefore, the findings of the current study did not portray the correct picture of the actual status. Therefore, the authors did not include the findings of BMI in the manuscript. The best method to assess the relationship between the overweight/obesity and oesophageal carcinoma would be to assess the overweight/obesity level prior to the onset of the disease, which is not feasible in a case-control study.

The Asian diet especially in South Asia is usually spicy and excessively coloured compared to other countries in the world. In the current study, frequent consumption of meals with excessive chillies was a potential risk factor in the univariate analysis. In concurrence, a study conducted in Assam, India showed similar results [26], while it was in contrast to the findings from Australia [49]. Also, consumption of food/beverages with excessive colouring was found to be a potential risk factor in the current study. This denotes the possibility of these factors being region specific risk factors for oesophageal carcinoma resulting in its high incidence rate. Therefore, the association of these factors with oesophageal carcinoma need to be further explored.

The association of high-risk alcohol consumption with oesophageal cancer demonstrated in the current study was in concurrence with other studies done in Western countries and China [18, 34]. Many studies globally have also shown that smoking is a risk factor for oesophageal carcinoma [25, 50]. However, though tobacco smoking was identified as a potential risk factor in the univariate analysis, the current study failed to show such a relationship following the multivariate analysis. High correlation with other risk factors such as alcohol consumption and betel chewing could be a reason for this result. On the other hand, detection of a significant association could have been limited by the small sample size of the current study. Despite strong evidence in the literature showing male sex as a risk factor for oesophageal cancer [1, 51], the current study failed to show such a relationship but possible effect modification by the male sex on the relationship between betel quid chewing and oesophageal carcinoma. This implies that the vulnerability of males for oesophageal cancer is only through their behavioural (e.g. alcohol consumption, betel chewing, poor diet quality), and environment and occupational risk factors (agrochemical exposure). This is an important finding for the planners of preventive programmes in the country, where the lifestyle modification especially among the males should be stressed upon. However, the small sample size of the current study urges the need for larger studies to assess the association of male sex and oesophageal carcinoma.

Noteworthy findings of this study are novel risk factors of oesophageal cancer identified in relation to occupation and environment. However, these findings especially on the exposure to agrochemicals were in contrast to many studies. A population based endoscopic survey conducted in the northern part of the Henan province, China, found that there is no association between the exposure to pesticides and oesophageal squamous cell carcinoma or its precursor lesion (OR = 1.0; 95% CI: 0.7, 1.4) [52]. A study conducted in Nebraska found that there is no association of adenocarcinoma with ever-use of insecticides (OR = 0.7; 95% CI: 0.4, 1.1) or herbicides (OR = 0.7; 95% CI: 0.4, 1.2) [53]. Since all these studies including our study have considered exposure to agrochemicals as a proxy measure, further exploration of this relationship using exact measurements of the pollutants in the environment is highly recommended.

With regards to the risk pertaining to drinking water source, a cohort study conducted in Linxian, China, found that the piped water was inversely associated with oesophageal carcinoma (OR = 0.86; 95% CI: 0.78, 0.96) [25]. A matched case-control study conducted in Iran also found that drinking un-piped water was a risk for oesophageal squamous cell carcinoma (OR = 4.25; 95% CI: 2.2, 8.1) and this risk increased with every 10 years of drinking un-piped water with an OR of 1.47 (95% CI: 1.2, 1.8) [54]. In contrast, pipe-borne water was seen to have a six-fold risk for oesophageal carcinoma in the present study compared to other sources of water. Although pipe-borne water supply is regulated at national level in Sri Lanka, and being treated through physical and chemical methods to improve the quality at the point of source, re-contamination could occur during the distribution to consumers owing to inadequacies of the infrastructure (e.g. leaking conveyances), and unsafe storage and handling practices of the consumer [55]. Also, it could be due to the contamination of water sources with heavy metals. A local study found that the largest source of drinking water in the district of Colombo is contaminated with heavy metals such as Tin, Zinc, Cobalt, Iron and Nickel [56]. Additionally, adjacent dug wells are reported to be contaminated with heavy metals such as Lead, Cadmium and Zinc, most likely due to pollution by industries established around these water sources [56]. Further exploration is therefore highly recommended on the relationship of heavy metal contamination of water with oesophageal carcinoma.

It is shown that oesophageal carcinoma is strongly related with old age [57]. Based on the available cancer incidence data for Sri Lanka, most cases of oesophageal carcinoma were in the 65–69 year age group for males, although a rise in its incidence was apparent after 40 years of age for both sexes [5, 39]. Therefore, 65 years was taken as the cut off for assessing age as a risk factor for oesophageal carcinoma in the current study, which demonstrated a significant association with oesophageal carcinoma. Also, the high risk of oesophageal cancer associated with family history of cancer is shown in many studies globally [58, 59] including the current study, highlighting the importance of family screening. These factors emphasise the significance of screening the high risk population for oesophageal carcinoma by UGIE. However, this may not be feasible in a low resource country like Sri Lanka owing to the associated prohibitive cost, unavailability of equipment and trained/skilled personnel. This points out the importance of developing risk prediction models based on the country specific risk factors, so that the individuals with substantial risk can be timely identified and referred for endoscopic examination to exclude oesophageal cancer.

### Strengths and limitations

Although the largest calculated sample size (based on the latest available local and international literature) was considered in the present study, the sample seemed not powered enough to detect a significant association with some of the potential risk factors (e.g. tobacco smoking and male sex) owing to small numbers in one or more of its categories. Therefore, larger studies to detect the association of these factors with oesophageal carcinoma is recommended.

The ideal control group for this study was apparently healthy persons recruited from the community who show no upper gastro-intestinal signs on UGIE. However, this was not possible as UGIE is not a routine screening procedure in hospitals and it is difficult to motivate an apparently healthy person to undergo it owing to the cost and invasiveness of the procedure. On the other hand, the mere absence of symptoms also cannot exclude oesophageal carcinoma, as symptoms develop only with substantial involvement of the tumour [60]. Thus, to avoid this misclassification bias, persons presenting with dyspeptic symptoms to the hospital for UGIE examination, had to be taken as controls. Not being apparently healthy, this control group may differ from the general population, as they may have higher exposure rates for the risk factors of oesophageal carcinoma such as alcohol consumption, consumption of deep fried food and spicy food. This possible sharing of risk factors might have resulted in a reduction in the magnitude of the risk of the risk profile identified for oesophageal carcinoma.

The current study was conducted in the Western province of Sri Lanka which is the most populated and the authors are aware that one may argue with the generalizability of the study findings. However, the Western province consists of the highest socio-economic diversity and include people from urban, rural and estate sectors [61]. Therefore, findings from the Western province related to risk factor profile is assumed not to differ much from the rest of the country. Nevertheless, the magnitude of the risk for a certain risk factor may differ for each province. Also, the sample size for the current study was calculated to capture the smallest risk reported in literature of the potential risk factors. Thus, even though there is a possibility of the risk magnitude to be differ for each province, the risk factor profile more or less could be generalizable to the country.

## Conclusions

The country specific risk factor model for oesophageal carcinoma includes novel and modifiable risk factors in relation to diet, occupation, environment and health related factors, in contrast to lesser risk associated with already known conventional risk factors. This emphasizes the need for developing community based targeted educational programmes and targeted preventive activities to combat the risk factors of oesophageal carcinoma for its primary prevention in Sri Lanka.

## Additional file

Additional file 1: STROBE Statement— for *case-control studies.* Description of data: STROBE statement checklist for the items to be included in reporting case-control studies. (DOCX 33 kb)

## Abbreviations

BMI: Body Mass Index
DALY: Disability Adjusted Life Years
FFQ: Food Frequency Questionnaire
LR: Logistic Regression
NCIM: National Cancer Institute, Maharagama
NSAIDs: Non-Steroidal Anti-Depressants
OR: Odds Ratio
SPSS: Statistical Package for the Social sciences
UGIE: Upper Gastro-Intestinal Endoscopy
YLD: Years Lived with Disability
YLL: Year of Life Lost
