# Supplementary material for: Novel risk factors for primary prevention of oesophageal carcinoma: a case-control study from Sri Lanka
Source: BMC Cancer. 2018 Nov 19;18:1135. doi: 10.1186/s12885-018-4975-4 (PMC6245903; doi:10.1186/s12885-018-4975-4)
Supplement: Supplementary file 1 — STROBE Statement— for case-control studies. Description of data: STROBE statement checklist for the items to be included in reporting case-control studies. (DOCX 33 kb) [file 12885_2018_4975_MOESM1_ESM.docx]

**Additional File 1.**

**STROBE Statement— for *case-control studies***

|  | Item No | Recommendation | Manuscript |
| --- | --- | --- | --- |
| **Title and abstract** | 1 | (*a*) Indicate the study’s design with a commonly used term in the title or the abstract | Title, Title page, page 1 |
|  |  | (*b*) Provide in the abstract an informative and balanced summary of what was done and what was found | Abstract, page 2 |
| Introduction | | |  |
| Background/rationale | 2 | Explain the scientific background and rationale for the investigation being reported | Background: page 3-6; rationale: page 5 – 2^nd^ paragraph and page 6 - 1st paragraph |
| Objectives | 3 | State specific objectives, including any prespecified hypotheses | Background, page 6, 1^st^ paragraph |
| Methods | | |  |
| Study design | 4 | Present key elements of study design early in the paper | Methods, page 6, 1^st^ paragraph |
| Setting | 5 | Describe the setting, locations, and relevant dates, including periods of recruitment, exposure, follow-up, and data collection | Duration and main setting: Methods, page 6, 1^st^ paragraph.  Setting for cases: Methods, page 6, 1^st^ paragraph. Setting for controls: Selection of controls section; page 7, 1^st^ paragraph. |
| Participants | 6 | (*a*) Give the eligibility criteria, and the sources and methods of case ascertainment and control selection. Give the rationale for the choice of cases and controls | Cases: Methods, page 6, Selection of cases section, 1^st^ paragraph  Controls: Methods, page 7, Selection of controls, 1^st^ paragraph. |
|  |  | (*b*) For matched studies, give matching criteria and the number of controls per case | N/A |
| Variables | 7 | Clearly define all outcomes, exposures, predictors, potential confounders, and effect modifiers. Give diagnostic criteria, if applicable | Diagnostic criteria for cases: Methods, page 6, Selection of cases section, 1^st^ paragraph.  Selection criteria for controls: page 7, selection of controls section, 1^st^ paragraph.  Study variables: Methods, page 8, 2^nd^ paragraph, study variables section. |
| Data sources/ measurement | 8* | For each variable of interest, give sources of data and details of methods of assessment (measurement). Describe comparability of assessment methods if there is more than one group | Methods, page 8, 3^rd^ paragraph, validated questionnaires used in the study section, explains the validated FFQ and LTPAQ used for assessing the quality of diet and the physical activity levels of the study participants.  Other variables: Methods, page 8, 4^th^ paragraph.  Same questionnaires were used to collect data from both case and control groups: Methods section, page 8, 2^nd^ paragraph, study variables section and 3^rd^ paragraph, validated questionnaires used in the study. |
| Bias | 9 | Describe any efforts to address potential sources of bias | Misclassification bias: Strengths and limitations, page 17, 2^nd^ paragraph.  Selection bias due to selection of cases and controls from two settings: Methods section, page 7, 2^nd^ paragraph. |
| Study size | 10 | Explain how the study size was arrived at | Methods, page 8, 1^st^ paragraph, sample size calculation section. |
| Quantitative variables | 11 | Explain how quantitative variables were handled in the analyses. If applicable, describe which groupings were chosen and why | Methods, page 9, 1^st^ paragraph, data analysis section. |
| Statistical methods | 12 | (*a*) Describe all statistical methods, including those used to control for confounding | Methods, page 9, 1^st^ paragraph, Data analysis section. |
|  |  | (*b*) Describe any methods used to examine subgroups and interactions | Methods, page 9, Data analysis section, 2^nd^ paragraph. |
|  |  | (*c*) Explain how missing data were addressed | N/A |
|  |  | (*d*) If applicable, explain how matching of cases and controls was addressed | N/A |
|  |  | (*e*) Describe any sensitivity analyses | Methods, page 9, 1^st^ paragraph, Data analysis section. |
| Results | | |  |
| Participants | 13* | (a) Report numbers of individuals at each stage of study—eg numbers potentially eligible, examined for eligibility, confirmed eligible, included in the study, completing follow-up, and analysed | Results, page 10, 1^st^ paragraph. |
|  |  | (b) Give reasons for non-participation at each stage | N/A |
|  |  | (c) Consider use of a flow diagram | N/A |
| Descriptive data | 14* | (a) Give characteristics of study participants (eg demographic, clinical, social) and information on exposures and potential confounders | Results, page 10, 2^nd^ paragraph and Table 2 |
|  |  | (b) Indicate number of participants with missing data for each variable of interest | N/A |
| Outcome data | 15* | Report numbers in each exposure category, or summary measures of exposure | Results, page 10, 4^th^ paragraph and Tables 3-5 |
| Main results | 16 | (*a*) Give unadjusted estimates and, if applicable, confounder-adjusted estimates and their precision (eg, 95% confidence interval). Make clear which confounders were adjusted for and why they were included | Results, page 10, 4^th^ paragraph and Tables 3-5.  Results, page 11, 1^st^ paragraph. |
|  |  | (*b*) Report category boundaries when continuous variables were categorized | Results, Table 3- Monthly family income; Table 4- Total lifetime occupation, household and sports and exercise activities. |
|  |  | (*c*) If relevant, consider translating estimates of relative risk into absolute risk for a meaningful time period | N/A |

| Other analyses | 17 | Report other analyses done—eg analyses of subgroups and interactions, and sensitivity analyses | Results, page 11, 1^st^ paragraph.  Interaction: Results, page 11, 2^nd^ paragraph. |
| --- | --- | --- | --- |
| Discussion | | |  |
| Key results | 18 | Summarise key results with reference to study objectives | Discussion, pages 11-12, 1^st^ paragraph. |
| Limitations | 19 | Discuss limitations of the study, taking into account sources of potential bias or imprecision. Discuss both direction and magnitude of any potential bias | Strengths and limitations, pages 17-18, 1-3 paragraphs. |
| Interpretation | 20 | Give a cautious overall interpretation of results considering objectives, limitations, multiplicity of analyses, results from similar studies, and other relevant evidence | Discussion, pages 12-17 |
| Generalisability | 21 | Discuss the generalisability (external validity) of the study results | Strengths and limitations, page 18, 1^st^ paragraph. |
| Other information | | |  |
| Funding | 22 | Give the source of funding and the role of the funders for the present study and, if applicable, for the original study on which the present article is based | Funding, page 20 |

*Give information separately for cases and controls.

**Note:** An Explanation and Elaboration article discusses each checklist item and gives methodological background and published examples of transparent reporting. The STROBE checklist is best used in conjunction with this article (freely available on the Web sites of PLoS Medicine at http://www.plosmedicine.org/, Annals of Internal Medicine at http://www.annals.org/, and Epidemiology at http://www.epidem.com/). Information on the STROBE Initiative is available at http://www.strobe-statement.org.
